# Decay of Sabin inactivated poliovirus vaccine (IPV)-boosted poliovirus antibodies

**DOI:** 10.1016/j.trivac.2015.08.001

**Published:** 2015

**Authors:** Sonia Resik, Alina Tejeda, Magile Fonseca, Carolyn Sein, Lai Heng Hung, Yenisleidys Martinez, Manuel Diaz, Hiromasa Okayasu, Roland W. Sutter

**Affiliations:** a Institute Pedro Kouri, Havana, Cuba; b Provincial Health Office, Camagüey, Cuba; c The World Health Organization, Geneva, Switzerland

**Keywords:** Adjuvant, Aluminum hydroxide, Inactivated poliovirus vaccine, Phase I trial, Sabin strains, Duration of antibodies

## Abstract

**Introduction:**

We conducted a follow-on study to a phase I randomized, controlled trial conducted in Cuba, 2012, to assess the persistence of poliovirus antibodies at 21–22 months following booster dose of Sabin-IPV compared to Salk-IPV in adults who had received multiple doses of oral poliovirus vaccine (OPV) during childhood.

**Methods:**

In 2012, 60 healthy adult males aged 19–23 were randomized to receive one booster dose, of either Sabin-inactivated poliovirus vaccine (Sabin-IPV), adjuvanted Sabin-IPV (aSabin-IPV), or conventional Salk-IPV. In the original study, blood was collected at days 0 (before) and 28 (after vaccination), respectively. In this study, an additional blood sample was collected 21–22 months after vaccination, and tested for neutralizing antibodies to Sabin poliovirus types 1, 2 and 3.

**Results:**

We collected sera from 59/60 (98.3%) subjects; 59/59 (100%) remained seropositive to all poliovirus types, 21–22 months after vaccination. The decay curves were very similar among the study groups. Between day 28 and 21–22 months, there was a reduction of ⩾87.4% in median antibody levels for all poliovirus types in all study groups, with no significant differences between the study groups.

**Conclusion:**

The decay of poliovirus antibodies over a 21–22-month period was similar regardless of the type of booster vaccine used, suggesting the scientific data of Salk IPV long-term persistence and decay may be broadly applicable to Sabin IPV.

## 1 Introduction

In 2008, the WHA recommended the WHO develop safer inactivated poliovirus vaccine (IPV) production technology using attenuated seed strains, such as Sabin polioviruses (Sabin-IPV) [1]. Sabin-IPV technology would partly address the biosafety risks associated with Sabin-IPV production, therefore allowing for production in developing countries [2], [3].

The immunogenicity of Sabin-IPV administered in the primary series has been well-established in different clinical studies in China, Japan, Poland, and Cuba [4], [5], [6], [7], some of which demonstrated that Sabin-IPV induced adequate neutralizing antibodies to both Sabin and wild poliovirus [6], [8]. Sabin-IPV products are currently licensed in Japan and China, and are under development in many other countries [9]. As Sabin-IPV and adjuvanted Sabin-IPV (aSabin-IPV) are expected to be widely used in the near future, it is important to assess the medium and long-term persistence of Sabin-IPV boosted antibody response.

Several studies have demonstrated the long-term presence of neutralizing antibodies, induced by Salk IPV [10], [11], [12], [13]. To date however, only one study has assessed the duration of immunity induced by Sabin-IPV. This was a phase III trial conducted in Japan, using tetravalent diphtheria-tetanus-acellular pertussis-Sabin-IPV vaccine (DTaP-Sabin-IPV), which demonstrated comparable immunity between Salk-IPV and DTaP-Sabin-IPV, 6–18 months after vaccination [5].

We conducted a follow-on study to the phase I Cuba study conducted in 2012. This study is the first to assess and compare the decay of neutralizing antibodies to poliovirus, between day 28 and 21–22 months, in adults who received multiple doses of oral poliovirus vaccine (OPV) during childhood, following a booster dose of either Sabin-IPV, aSabin-IPV, or Salk-IPV, in a tropical setting.

## 2 Methods

In the phase I trial in Cuba conducted in 2012, sixty healthy male subjects aged 19–23 years, who had received polio vaccination with multiple doses of OPV during childhood, in accordance with the Cuban national immunisation program, and with no history of receiving poliovirus vaccine since the age of 9 years, were enrolled and randomized, to receive a booster dose of either conventional Salk-IPV, or Sabin-IPV, or aSabin-IPV (adjuvanted with Aluminum hydroxide), with the following formulations: Salk-IPV 40:8:32 D-antigen Units per dose (DU/dose), Sabin-IPV 20:32:64 DU/dose, and aSabin-IPV 10:16:32 DU/dose and 0.5 mg aluminum hydroxide, respectively [14]. All Sabin-IPV and Salk-IPV vaccines were provided by the Netherlands Vaccine Institute (NVI) (currently called Intravacc) [7].

In our follow-on study, all subjects were contacted at 21–22 months after initial vaccination, for blood collection. Sera were tested at the Institute Pedro Kouri, for neutralizing antibodies to Sabin poliovirus types 1–3, using standard micro-neutralization assay. The antibody titers were diluted to 1:65,536, above the standard 1:1024 because high titers were expected with a boosting dose of IPV. Seroconversion was defined as a ⩾fourfold increase in reciprocal antibody titers.

We calculated the decay in antibody titers at day 28 and at 21–22 months, and the overall increase in antibody titers during day 0 and 21–22 months, by poliovirus type and study arm with 95% confidence intervals using bootstrapping sampling and estimation with 10,000 replications. We tested differences in antibody titers between the study groups, with Salk-IPV as the reference group, by poliovirus type, using Wilcoxon rank sum test with significance indicated by *p* ⩽ 0.05. All analyses were conducted using statistical application “R 3.1.2” [15].

## 3 Results

### 3.1 Study population

In the previous study, there were no significant differences between the three groups, in baseline characteristics of age, height, weight, time since receiving last OPV dose, or, baseline titer of neutralizing antibodies to Sabin poliovirus types 1–3 [7].

In our study, a total of 59/60 (93.1%) subjects were followed-up at 21–22 months (654–675 days). One subject in the aSabin-IPV arm was lost to follow-up, and one subject in the aSabin-IPV group had moved to Havana, where their blood was collected.

### 3.2 Antibody decay (day 28 to 21–22 months)

**Fig. 1:**
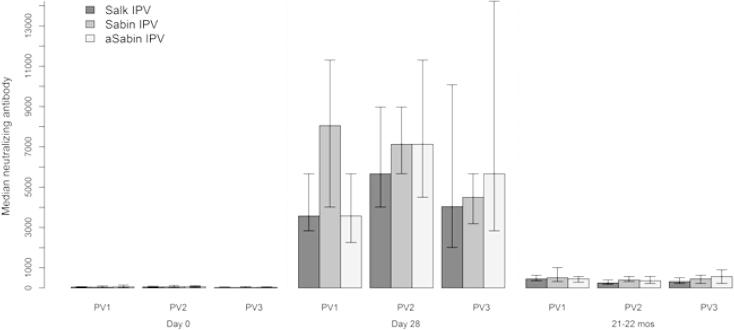
Median antibody titers (log_2_) to poliovirus types 1–3, on day 0, day 28, and 21–22 months, by study group; 95% confidence intervals calculated using bootstrapping with 10,000 replications.

In the previous study, there were no differences in immunogenicity to Sabin poliovirus types 1–3 between the study groups during day 0 and day 28, with all subjects seroconverting or boosted by day 28 [7]. At day 28, median antibody titers were highest for poliovirus type 1 in the Sabin-IPV study group, and for poliovirus types 2 and 3 in the aSabin-IPV group (Fig. 1).

In our study, at 21–22 months, all subjects had detectable antibody titers for all Sabin poliovirus types, with median antibody titers highest for poliovirus types 1 and 2 in the Sabin-IPV study group, and for poliovirus type 3, in aSabin-IPV group. Median titers were lowest for all poliovirus types in the Salk-IPV group. We did not find any significant differences in median antibody titers between the study groups for all poliovirus types (Fig. 1, Table 1).

There were no statistically significant differences in the decay of antibody titers during day 28 and 21–22 months, between Salk-IPV, Sabin-IPV and aSabin-IPV groups, with relative reduction as a percentage decline in median antibody titers by poliovirus type: 92.1%, 92.1%, 87.4% for poliovirus type 1 (*p* = 0.54; *p* = 0.61, respectively); 96.0%, 95.0%, 95.0% for poliovirus type 2 (*p* = 0.66; *p* = 0.93, respectively); 93.7%, 92.1%, 93.7% for poliovirus type 3 (*p* = 0.67; *p* = 0.50, respectively) (Table 2).

### 3.3 Antibody increase (day 0 to 21–22 months)

There were no statistically significant differences in the relative increase in antibodies from day 0 to 21–22 months between Salk-IPV, Sabin-IPV and aSabin-IPV study groups by poliovirus type: 1121.3%, 1028.5%, 893.0%, for poliovirus type 1 (*p* = 0.53; *p* = 0.40, respectively); 458.7%, 694.7%, 402.8% for poliovirus type 2 (*p* = 0.71; *p* = 0.95, respectively); 320.5, 450.0, 566.0, for poliovirus type 3 (*p* = 0.72; *p* = 0.54, respectively) (Table 2).

## 4 Discussion

This study is the first to assess the decay of neutralizing antibodies to Sabin poliovirus beyond 28 days after a booster dose of Salk-IPV, Sabin-IPV, and aSabin-IPV, and found no differences between the study groups in adults who received multiple doses of OPV during childhood.

The only other study assessing the decay of neutralizing antibodies to Sabin poliovirus beyond 28 days after vaccination, was conducted by Okada in Japan, however this assessed antibody titers induced in infants, 6–18 months following a primary series administration with three doses of combination DTaP-Sabin-IPV; the relative percentage decline in median antibody titers during day 28 and 6–18 months was 75.3%, 63.9%, 86.3%, for poliovirus types 1–3, respectively [8].

The greater decay for Sabin-IPV may have been due to higher median antibody titers boosted by Sabin-IPV at day 28 with baseline of multiple OPV doses in our study, compared to lower median antibody titers induced by DTaP-Sabin IPV at day 28 following primary schedule, in Okada’s study: 3565.8, 5792.6, 4096, for Sabin-IPV in our study, compared to 2076.6, 1428.2 and 1663.5, induced by DTaP-Sabin-IPV in Okada’s study for poliovirus types 1, 2 and 3, respectively. However, the greater decay observed may have been an artifact, due to the differences in the dilution of antibody titers; in our study antibody titers were diluted up to 1:65,536, however we were not able to verify the level of dilution in Okada’s study.

Previous boosting studies for Salk-IPV demonstrated greater antibody decay following booster dose in the developing country, compared to industrialized country setting. A study conducted in Oman by Sutter demonstrated a relative reduction of 72.4% in median antibody titers to poliovirus type 3, 6 months after administration of a booster dose of Salk-IPV, in children who received a primary schedule of OPV. Furthermore, the decay in median antibody titers may have been greater in this study, as antibody titers were only diluted up to the standard 1:1448 [15]. This contrasts with findings from the Netherlands, published by Rumke, which demonstrated a relative reduction in antibody geometric mean titers of 62.1%, 71.3%, 83.5% to poliovirus types 1, 2, and 3, respectively, 5 years after administration of a booster dose of DT-Salk-IPV, in children who received a primary schedule of DTP-IPV, and a booster dose of DT-IPV at 4 years [16], [17].

Our study had some limitations. The sample size was small, limited to healthy adults from a potentially socio-economically homogenous area. The study was conducted in a specific tropical developing country setting. While our study found similar rates of antibody decay in adults, at 21–22 months between Sabin-IPV, aSabin-IPV, and Salk-IPV groups, we cannot directly extrapolate this trend to longer term decay between the groups. Therefore similar studies are needed to assess and compare medium and long term antibody decay, particularly in naive children.

Our study only assessed antibodies against Sabin virus type 1, 2, 3. Previously a study conducted in infants in Poland using the same vaccine (produced by Intravacc), demonstrated that seroconversion rates against both Sabin and wild virus were equivalent between the Sabin-IPV and Salk-IPV groups; however, neutralizing antibody titers induced by Sabin-IPV were higher against Sabin strains compared to wild poliovirus strains, and similarly, titers induced by Salk-IPV were higher against wild poliovirus strains [18]. Therefore, although we assume the rate of decay of antibodies against Salk strains is similar between Salk and Sabin IPV, the cross neutralisation effect is worth assessing in future studies.

There may have been secondary exposure to OPV during the study period (10/2012–08/2014); however we concluded that secondary exposure of enrolled subjects was minimal, as although there were four national OPV campaigns targeting children under 3 years of age, two of which also included children 9 years of age, during this period, we did not find that any subjects demonstrated an increase in antibody titers between day 28 and 21–22 months.

This study demonstrates comparable immunogenicity and trends in antibody decay 21–22 months in adults who had received multiple doses of OPV, after booster dose Sabin-IPV and aSabin-IPV, to Salk-IPV. This should galvanize momentum for countries and vaccine producers to further develop Sabin-IPV and aSabin-IPV technology. Future research should be conducted to further assess, and build the evidence for the medium and long-term immunity of Sabin-IPV.

## Role of the funding source

Funding for this study was provided by World Health Organization, Geneva and the Ministry of Health, Labour and Welfare, Japan. Intravacc provided inactivated poliovirus vaccines free of charge for this study.

## Role of medical writer or editor

No medical writer or editor was engaged.

## Ethics committee approval

This study was approved by the Ethics Review Committee of the World Health Organization in Geneva, by the Ethics Review Committees of the Pedro Kouri Institute in Havana, and the Provincial Center for Hygiene and Epidemiology, Camaguey.

## Declaration of interests

Authors did not declare any conflict of interest.

## Figures and Tables

**Table 1:** Median antibody titers (log_2_) to poliovirus types 1–3, on day 0, day 28, and 21–22 months, by study group with 95% confidence intervals calculated using bootstrapping with 10,000 replications.

		Day 0		Day 28		21–22 months	
Median titer (95% CI)	*p*-Value	Median titer (95% CI)	*p*-Value	Median titer (95% CI)	*p*-Value
Type 1	Salk IPV	45.0 (18.0–71.0)	Ref	3573.0 (2839.0–5664.0)	Ref	450.0 (357.0–635.3)	Ref
Sabin IPV	36.0 (25.4–100.8)	0.322	8053.0 (4009.4–11300.0)	0.271	508.0 (318.4–1007.3)	0.693
aSabin IPV	57.0 (14.0–142.0)	0.473	3573.0 (2255.0–5664.0)	0.365	450.0 (284.0–566.0)	0.64

Type 2	Salk IPV	51.0 (28.0–90.0)	Ref	5664.0 (4009.4–8976.0)	Ref	254.5 (179.0–400.8)	Ref
Sabin IPV	57.0 (36.0–126.9)	0.957	7130.0 (5664.0–8976.0)	0.225	403.5 (318.4–566.0)	0.177
aSabin IPV	71.0 (36.0–113.0)	0.693	7130.0 (4499.0–11300.0)	0.365	357.0 (225.0–566.0)	0.352

Type 3	Salk IPV	25.5 (15.9–50.6)	Ref	4036.0 (2009.7–10071.2)	Ref	320.5 (225.0–504.7)	Ref
Sabin IPV	25.5 (14.1–71.0)	0.755	4499.0 (3184.9–5664.0)	0.946	450.0 (225.0–635.3)	0.724
aSabin IPV	28.0 (14.0–57.0)	0.921	5664.0 (2839.0–14226.0)	0.248	566.0 (225.0–898.0)	0.535

*Note:* excluded 1 subject missing data for 21–22 months.

**Table 2:** Relative increase in median antibody titers (%) between day 0 and 21–22 months; relative reduction in median antibody titers during day 28 and 21–22 months, to poliovirus types 1–3, by study group with 95% confidence intervals calculated using bootstrapping with 10,000 replications.

		Day 0 and 21–22 months comparison		Day 28 and 21–22 months comparison	
Median % relative increase (95% CI)	*p*-Value	Median % relative reduction (95% CI)	*p*-Value
Type 1	Salk IPV	1121.3 (896.4–2126.5)	Ref	92.1 (84.2–92.9)	Ref
Sabin IPV	1028.5 (535.1–1714.3)	0.534	92.1 (90.0–93.7)	0.543
aSabin IPV	893.0 (151.4–1904.2)	0.399	87.4 (80.1–92.1)	0.613

Type 2	Salk IPV	458.7 (147.3–1032.1)	Ref	96.0 (92.9–96.8)	Ref
Sabin IPV	694.7 (124.4–1305.0)	0.705	95.0 (93.7–96.0)	0.655
aSabin IPV	402.8 (100.0–893.0)	0.945	95.0 (93.7–96.8)	0.933

Type 3	Salk IPV	1043.9 (685.8–1670.5)	Ref	93.7 (91.0–95.5)	Ref
Sabin IPV	1276.9 (250.1–2727.8)	0.892	92.1 (91.0–95.0)	0.665
aSabin IPV	1150.0 (694.7–2396.5)	0.768	93.7 (92.1–96.8)	0.500

*Note:* excluded 1 subject missing data for 21–22 months.
